# Intraoperative indocyanine green with ultrasound-guided localization as prodigious adjuncts explicating negative margin in functional insulinoma: A case report

**DOI:** 10.1016/j.ijscr.2024.110805

**Published:** 2024-12-28

**Authors:** Prabir Maharjan, Sneha Raut, Suman Paudel, Arika Baral, Dhiresh Kumar Maharjan, Prabin Bikram Thapa

**Affiliations:** a Department of Gastrointestinal and General Surgery, Kathmandu Medical College and Teaching Hospital, Kathmandu, Nepal; b MBBS, Kathmandu Medical College and Teaching Hospital, Kathmandu, Nepal; c Department of Radiology and Radiodiagnosis, Kathmandu Medical College and Teaching Hospital, Kathmandu, Nepal; d Department of Pathology, Kathmandu Medical College and Teaching Hospital, Kathmandu, Nepal

**Keywords:** C-peptide, Insulinoma, Intraoperative fluorescence, Pancreatic neuroendocrine neoplasm, Pancreaticoduodenectomy

## Abstract

**Introduction and importance:**

Insulinomas are rare pancreatic neuroendocrine neoplasms with an incidence of one to four cases per million annually and a 5 % to 10 % association with hereditary multiple endocrine neoplasia type-1. While most insulinomas are benign and well-encapsulated, approximately 6 % may have malignant potential. Intraoperative localization remains a vital component of treatment, often facilitated by modern imaging techniques like intraoperative ultrasound and fluorescence modalities.

**Case presentation:**

A 52-year-old woman was referred to Kathmandu Medical College with generalized weakness, recurrent headaches, and fatigue relieved by food intake. She had a history of hypoglycemia-induced abnormal body movements and loss of consciousness. After biochemical and imaging evaluations, she was diagnosed with pancreatic insulinoma. Based on the higher affinity of neuroendocrine tumoral cells for Indocyanine Green compared to normal pancreatic cells, the patient underwent Indocyanine Green-directed laparoscopic-assisted pancreaticoduodenectomy managed perioperatively with subcutaneous octreotide. She had an uneventful postoperative period and was discharged on the eighth day.

**Discussion:**

Insulinomas present a unique diagnostic and therapeutic challenge, especially in cases of sporadic occurrence. Surgical resection is the mainstay of treatment, with enucleation preferred for benign tumors. In this case, fluorescence-guided surgery and intraoperative ultrasound aided in accurate localization and successful excision.

**Conclusion:**

Insulinomas, though rare, require prompt diagnosis and surgical intervention to prevent malignancy and metastasis.

## Highlights

• Intraoperative ICG & ultrasound aid in insulinoma localization & excision.
• Fluorescence-guided surgery optimizes treatment for functional insulinoma.
• Early detection & advanced imaging enhance surgical outcomes in insulinomas.

## 1 Introduction

Neuroendocrine tumors (NETs) of the pancreas are classified into well-differentiated, poorly-differentiated, and mixed neuroendocrine-non-neuroendocrine neoplasms (WHO 2017) [1]. Typically benign and solitary, they can occur sporadically or as part of syndromes like MEN I and von Hippel-Lindau [1]. More than 90 % of insulinomas are benign, small, well-encapsulated solitary tumors [1]. However, 5 %–10 % are associated with hereditary syndromes, and 6 % have malignant potential [2]. The challenge of diagnosis is that 10 % to 27 % of these remain undetected if preoperative localization is not considered [2].

Assessment of the extent of pancreatic tumor is mainly done by inspection and palpation and with intraoperative ultrasonography, however even with these modalities the identification of the extent is challenging because of inflamed surrounding pancreatic tissue [2,3]. Insulinomas present with different clinical symptoms, classically with repeated episodes of hypoglycemia [3]. Neurologic dysfunction and neuroglycopenic symptoms usually follow severe hypoglycemia (<50 mg/dl): confusion, visual disturbances, loss of consciousness, seizures, or rarely focal neurologic deficits resembling a stroke [3]. Neuroglycopenic symptomatology should be considered a “red flag” that should prompt suspicion and evaluation for hyperinsulinemia [3]. Biochemical testing for plasma glucose levels <45-50 mg/dl, elevated insulin level > 6μU/ml, and C-peptide levels ≥200 pmol/l with negative screening for sulphonylurea is ideal [3]. Diagnostic imaging modalities include Computed tomography (CT), Magnetic Resonance Imaging (MRI) of the abdomen and pelvis, Endoscopic ultrasound, and Glucagon-like peptide- 1 receptor (GLP-1R) [3]. Surgical removal is typically curative for insulinomas; however, localization remains challenging due to their small size (<2 cm) [4]. Conventional imaging techniques, such as transabdominal ultrasound, contrast-enhanced CT, and MRI, demonstrate variable detection rates of 9–63 %, 63–94 %, and 60–90 %, respectively [4]. Intraoperative ultrasound (IOUS) provides superior accuracy (>90 %) by detailing tumor location and its relationship to the pancreatic duct [5]. Advanced techniques like ICG fluorescence further enhance localization by improving intraoperative visualization, offering a valuable adjunct to standard methods [5].

Surgeries that preserve the pancreas are favored as much as possible while addressing insulinoma. Since most are benign and solitary, tumor enucleation is advised whenever possible [6]. However, the size of insulinoma of >2 cm and <2 mm proximity with the main pancreatic duct are contradictory characteristics to performing Whipple or distal pancreatectomy surgeries [6].

Herein, we present a case of a patient with a sporadic insulinoma successfully managed with laparoscopic-assisted tumor excision, highlighting the diagnostic and therapeutic challenges associated with this condition.

The work has been reported in line with the SCARE criteria 2023 [7].

## 2 Case presentation

A 52-year-old lady was referred to Kathmandu Medical College and Teaching Hospital, with a history of generalized weakness, recurrent headaches, and fatigue, which improved with frequent meals. The patient also gave a history of blurred vision, loss of attention, abnormal body movements, and loss of consciousness a few years back. Evaluated at various centers for recurrent hypoglycemia over the past year, she was recently hospitalized for 8 days for hypoglycemia, with levels recorded as low as 17 mg/dl, managed with intravenous dextrose.

**Fig. 1:**
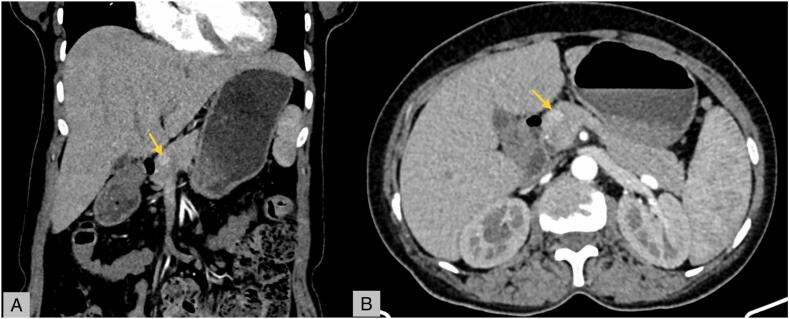
(A) CT Pancreatic protocol: Arterial phase – coronal view, (B) CT pancreatic protocol: Arterial phase – axial view.

The contrast-enhanced computed tomography (CECT) revealed a 12 mm × 11.9 mm isodense lesion in the head of the pancreas, showing avid enhancement on the arterial phase and peripheral enhancement on the portal phase, adjacent to the main pancreatic duct, suggesting the diagnosis of neuroendocrine tumor (Fig. 1).

Blood tests showed high C-peptide (>0.2 nmol/l) and insulin levels (>6 μIU/ml) with blood glucose (<50 mg/dl) at 34 mg/dl, insulin at 21 μIU/ml, and C-peptide above 0.6 nmol/l. Financial constraints precluded further advanced investigations such as Proinsulin, sulphonyl urea, Endoscopic ultrasound, positron emission tomography scan, or MRI. The patient subsequently underwent laparoscopic-assisted pancreaticoduodenectomy.

### 2.1 Therapeutic intervention

**Fig. 2:**
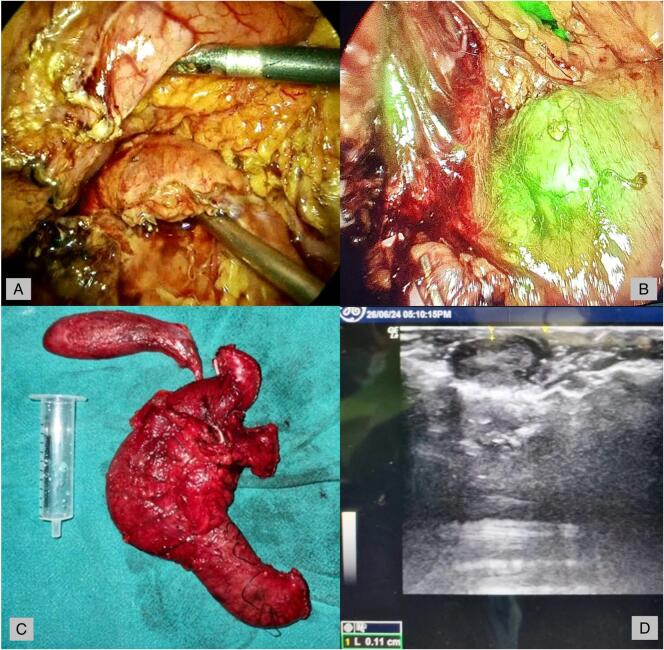
(A) Laparoscopic identification of pancreatic head insulinoma, (B) confirmation of the insulinoma in the pancreatic head after NIF-ICG fluorescence, (C) specimen of pancreaticoduodenectomy for pancreatic head insulinoma, (D) intraoperative ultrasound of the pancreatic head insulinoma.

Intravenous dextrose was administered the night before surgery to prevent fasting hypoglycemic episodes. Intraoperatively, the blood glucose was monitored every 15–30 min, and bolus doses of dextrose were given only when the glucose levels dropped down below 40 mg/dl. Intraoperative ultrasound and near-infrared fluorescence imaging with indocyanine green (NIF-ICG) localized the lesion at the pancreatic head (Fig. 2).

Two boluses of 7.5 mg (25 mg/10 ml) ICG intravenously and intralesional 5 mg ICG were administered, totaling 25 mg intraoperatively. The fluorescence after 5 min of each bolus showed a lesion of approximately 1.5 × 1 cm^2^ localized at the head of the pancreas, however, due to proximity to the main pancreatic duct, the patient underwent laparoscopic-assisted pancreaticoduodenectomy. The duration of the surgery was 310 min. The patient was observed in the surgical ICU for three days. Perioperative intravenous octreotide 100 μg injections were given, and the patient was discharged on the eighth postoperative day.

### 2.2 Pathology findings

**Fig. 3:**
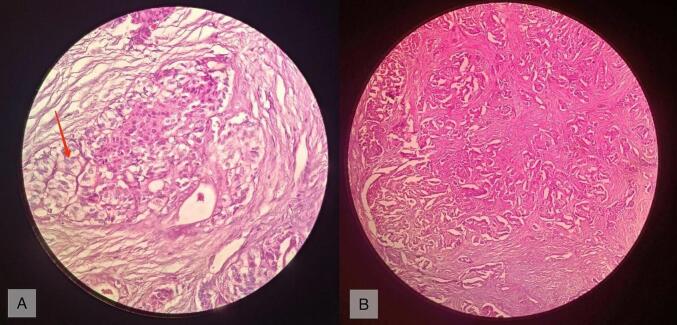
(A) Neuroendocrine tumor showing stippled chromatin (red arrow), (B) well differentiated neuroendocrine tumor arranged in trabeculae, glandular and pseudoglandular pattern embedded in desmoplastic stroma (H and E, low power). (For interpretation of the references to colour in this figure legend, the reader is referred to the web version of this article.)

Histopathology report revealed a unifocal 12 × 8 mm^2^, Grade 1, well-differentiated pancreatic neuroendocrine tumor with a mitotic rate of 0–1 per 10 HPF (2 mm^2^) without any lymphatic, vascular, or perineural invasion (Fig. 3).

The patient's immunohistochemistry panel showed immunopositivity for Synaptophysin, Chromogranin (strong and diffuse), and Pan-CK (weak) with a final diagnosis of well-differentiated neuroendocrine tumor (Functional Insulinoma).

### 2.3 Follow-up

The postoperative course was uneventful. Follow-up in insulinoma is not done frequently unless and until it is associated with MEN-1 syndrome or possesses malignant potential. A comprehensive follow-up plan is critical for patients who have undergone laparoscopic-assisted pancreaticoduodenectomy for pancreatic head insulinoma to monitor for recurrence, manage complications, and assess long-term outcomes.

In this case, the patient was observed closely in the initial weeks. Short-term follow-ups every three days for two weeks were conducted to monitor immediate postoperative recovery and glycemic control. Weekly evaluations over the next two weeks ensured stabilization, and monthly follow-ups were scheduled for the next three months to assess long-term outcomes. These follow-ups included a thorough clinical evaluation, fasting blood glucose levels, and serum insulin assessments to detect hypoglycemia or recurrence. Imaging studies, such as contrast-enhanced CT or MRI, were advised annually or earlier if symptoms suggestive of recurrence emerged.

This structured follow-up approach was tailored to mitigate the risk of recurrence and optimize the patient's recovery trajectory. The patient was counselled on recognizing signs of recurrence, maintaining regular follow-ups, and adhering to dietary recommendations to avoid hypoglycemic episodes.

## 3 Discussion

Patients with insulinoma characteristically present with the Whipple triad, which includes documented hypoglycemia (<45-50 mg/dl), neuroglycopenic symptoms, and resolution of the symptoms with glucose administration [8]. This triad, first described by Professor Allen Whipple in 1938, was observed in our patient [8].

Medical intervention can prevent hypoglycemia before surgery or when surgery is not feasible [9]. Diazoxide (50–300 mg/d with a maximum dose of 600 mg/d) is commonly used, suppressing insulin secretion and enhancing glycogenolysis, often combined with small, frequent meals to prevent hypoglycemia [9].

Boukhman MP et al. shared their experience from 1950 to 1995 on their patients with insulinoma. Over 41 years, a total of 68 surgeries were performed, 11 of those patients had MEN 1 syndrome, and seven with multiple insulinomas [1]. About 15 % had malignant insulinomas, 44 % underwent enucleation, and 64 % had distal pancreatectomy [1]. When intraoperative tumor localization was difficult, they suggested obtaining pancreatic vein sampling for insulin analysis or distal pancreatectomy [1,10,11].

Paiella et al. demonstrated that intravenous injection of ICG leads to better visualization of neuroendocrine with a mean latency time of 80 s and a mean visibility time of 220 s with the peak visualization of the tumor at 20 min after the administration of the last ICG bolus [12]. A recent retrospective single-arm cohort study by Haisu et al. highlighted the utility of indocyanine green (ICG) dynamic perfusion in evaluating abnormal vascular perfusion in tumors, offering functionality comparable to intraoperative real-time angiography [5]. The study suggests that injecting ICG beneath the tumor pseudocapsule could serve as an effective technique for achieving real-time, three-dimensional demarcation, thereby facilitating precise resection of insulinomas [5].

North American Neuroendocrine Tumor Society (NANETS), the European Neuroendocrine Tumor Society (ENETS), the European Society of Medical Oncology (ESMO), and the National Comprehensive Cancer Network (NCCN) have established their specific guidelines for neuroendocrine tumors [13,14]. ESMO guidelines 2020 recommend surgery for young patients, local or locoregional disease or NET of G1 and G2 grade, especially for functioning neoplasms irrespective of tumor size [14]. Watch-and-wait strategies have been recommended for asymptomatic neuroendocrine tumors of 2 cm with a short follow-up, especially for the elderly group of patients [13,14]. ESMO recommends enucleation as an alternative approach in selected patients especially for solitary, functioning 2 cm insulinomas and the necessity of >2 mm distance from the main pancreatic duct [13,14].

Similarly, ENETS recommends standard pancreatectomy for NET >2 cm with regional lymphadenectomy [15]. The NCCN recommendations are also similar with smaller lesions to be either observed or enucleated and larger lesions to either undergo pancreaticoduodenectomy or a distal pancreatectomy according to their location at the pancreas [15].

Complete surgical resection is the recommended treatment, being curative in most cases [2]. Preoperative localization is imperative as it optimizes surgical planning, with noninvasive imaging techniques such as ultrasound, CT, and MRI being beneficial [2]. MRI is however superior to CT in detecting metastasis before surgery. Our patient underwent only CT abdomen and pelvis due to financial constraints, but intraoperative use of NIF-ICG fluorescence was utilized for intraoperative localization of the pancreatic insulinoma [2,16].

ICG fluorescence imaging, initially utilized for surgical navigation and localization of liver tumors, is now applied for pancreatic malignancies [17]. If the ICG is given intravenously also known as negative staining, it reveals the vascular perfusion of tumors, eventually localizing the lesion [17]. Given its 2–3 s dynamic perfusion, multiple ICG boluses can accurately identify the lesion [17]. In our patient, intraoperatively we administered repeated bolus of ICG to visualize the lesion. Intraoperative ultrasonography is another adjunct for localization that, if combined with ICG can give favorable synergistic outcomes, especially for occult insulinomas [17].

## 4 Conclusion

Understanding the molecular mechanism driving the onset and spread of this illness will improve the prognosis, therapy, and care of benign and malignant insulinomas. Nevertheless, we have become more adept at identifying and treating insulinomas. With more recent and sophisticated biochemical evaluations, an earlier and more precise diagnosis would be possible. Furthermore, improved localization studies could lead to even higher surgical cure rates. The number of successful resections has increased as a result of upgraded laparoscopic techniques including adjuncts like NIF-ICG Fluorescence and Intraoperative Ultrasonography.

## Author contribution

All authors contributed to the study's conception and design. Material preparation and data collection were performed by P.M., S.R., P.G., S.P., A.B. The first draft of the manuscript was written by P.M. and S.R. and all authors commented on previous versions of the manuscript. All authors read and approved the final manuscript.

## Informed consent

The patient and the respective legal guardians were informed about the publication and provided informed consent before the reporting.

## Ethical approval

Our institution does not require ethical approval for reporting individual cases.

## Guarantor

Dr. Prabir Maharjan.

## Research registration number

None.

## Funding

None.

## Conflict of interest statement

The author(s) declared no potential conflicts of interest with respect to the research, authorship, and/or publication of this article.
